# “Letting themselves go during care” – exploring patient autonomy during co-designed intrapartum care in a Beninese maternity ward

**DOI:** 10.1186/s12884-024-06777-5

**Published:** 2024-08-30

**Authors:** Nicole S. Rodriguez Neufeld, Christelle Boyi Hounsou, Armelle Akouavi Vigan, Regine Unkels, Gisèle Houngbo, Alice Stockart, Claudia Hanson, Jean-Paul Dossou, Helle Mölsted Alvesson

**Affiliations:** 1 Department of Global Public Health, Karolinska Institutet, Stockholm, Sweden; 2 Centre de Recherche en Reproduction Humaine Et en Démographie, Cotonou, Benin

**Keywords:** Patient autonomy, Respectful maternity care, Experience of childbirth, Sub-Saharan Africa, Alternative care practices, Spiritual care

## Abstract

**Background:**

Patient autonomy is central to the provision of respectful maternity care. Enabling women to make decisions free of discrimination and coercion, and respecting their privacy and confidentiality can contribute to positive childbirth experiences. This study aimed to deepen the understanding of how patient autonomy is reflected through social practices during intrapartum care in Benin.

**Methods:**

Semi-structured interviews with women and midwives, a focus-group discussion with women’s birth companions, and non-participant observations in the delivery room were conducted within the frame of the ALERT research project. This study analysed data through a reflexive thematic analysis approach, in line with Braun and Clarke.

**Results:**

We identified two themes and five sub-themes. Patient autonomy was systemically suppressed over the course of birth as a result of the conditions of care provision, various forms of coercion and women’s surrendering of their autonomy. Women used other care practices, such as alternative medicine and spiritual care, to counteract experiences of limited autonomy during intrapartum care.

**Conclusions:**

The results pointed to women’s experiences of limited patient autonomy and their use of alternative and spiritual care practices to reclaim their patient autonomy. This study identified spiritual autonomy as an emergent dimension of patient autonomy. Increasing women’s autonomy during childbirth may improve their experiences of childbirth, and the provision of quality and respectful maternity care.

**Supplementary Information:**

The online version contains supplementary material available at 10.1186/s12884-024-06777-5.

## Background

Respecting patient’s autonomy during childbirth is central to the provision of respectful and rights-based maternity care [1–4]. In accordance with sexual and reproductive health and rights (SRHR), individuals have the right to unrestrained access to the information, goods and services needed to freely make decisions concerning their bodies and their sexual and reproductive health [5]. On a similar note, respectful maternity care (RMC) is defined as “care organized for and provided to all women in a manner that maintains their dignity, privacy and confidentiality, ensures freedom from harm and mistreatment, and enables informed choice and continuous support during labour and childbirth” [6], and is recommended for a positive childbirth experience [6, 7]. Respectful and patient-centred communication has been associated with improvements in pain management and emotional well-being and, on a societal level, with a decrease in maternal morbidity and mortality [8]. Despite global increases in accessibility and use of maternal health services, maternal mortality has not decreased sufficiently to reach global public health targets [2, 3]. This access-outcome gap can be attributed to the provision of sub-standard healthcare services [9]. With a rate of 545 maternal deaths per 100 000 live births in Sub-Saharan Africa (SSA) in 2020, improving the provision of respectful and quality maternity care in this region is, therefore, of utmost importance [9, 10].

Autonomy is integral to multiple aspects of RMC provision, such as effective communication and respecting women’s choices [2, 11]. The concept of patient autonomy has mainly been developed in acute healthcare contexts, and from a legal perspective, due to healthcare providers’ need to make medical decisions ethically and quickly [12, 13]. Autonomy during care has, therefore, widely been equated to decisional autonomy in the literature [12, 13], and is grounded in the concepts of liberty from controlling influences and agency [12, 14]. Although this conceptualization is central to ensuring informed consent, it may lead to reducing patients to a “thing with rights” [11]. The multidimensional framework for patient autonomy, developed by Arrieta Valero in the context of chronic care provision, argues that patient autonomy is a relational concept [12]. As such, it acknowledges the socio-environmental surroundings in which a patient will exert their autonomy and the way in which interactions between healthcare providers and patients can shape this autonomy. Arrieta Valero argues that patient autonomy emerges in light of the new identity a person will assume through their interactions with healthcare providers, family and society during care, and the change in their relationship to their bodies over the course of illness [12]. The change in a patient’s relationship to their body may similarly occur through other mechanisms, such as pregnancy. This comprehensive framework proposes five dimensions of autonomy, namely decisional, functional, executive, narrative and informational [12, 14]. We used this framework to guide our understanding of the complex manifestations of autonomy during intrapartum care in a Beninese maternity ward.

Several tools for the assessment of RMC, with autonomy as a sub-component, have previously been developed and applied in practice [7]. Disagreements in the literature on the conceptual definition of autonomy, a lack of consistent and transparent use of the terminology [15] and the inherent uncertainties of the progression of childbirth complicate the assessment of autonomy during childbirth. In some studies a woman’s level of empowerment within her community, and decision-making power within and outside of the healthcare context, is used as a measure of autonomy [15]. In other studies, autonomy has been reduced to informed decision-making or bodily autonomy [7]. In addition, healthcare providers have a responsibility for the life of the mother and her child simultaneously, which poses a challenge in how the concept of autonomy is applied in practice [7]. In light of the aforementioned conceptual and practical complexities around autonomy during maternity care, in addition to the influence of social contexts on interactions between women, healthcare providers and society, assessing and comparing women’s experience of autonomy during childbirth across various global contexts poses a great challenge.

Research on intrapartum care in SSA has extensively focused on women’s experiences of intrapartum care provision, and the influence of women’s societal autonomy on their utilization of maternal health services [15–20]. Access to and usage of intrapartum care services is influenced by several social determinants, such as socioeconomic status, distance to healthcare facilities, and power dynamics within the family and society [9, 20, 21]. It has been reported that many women experience sub-standard institutional intrapartum care, exemplified by accounts of mistreatment and limited communication by healthcare providers [2, 9, 22]. Mistreatment during childbirth has been reported to be common, regardless of its form and the context in which it occurs [7]. Although many non-clinical components of quality intrapartum care are usually relatively inexpensive, such as providing birthing women with emotional support, they are not widely implemented or equally prioritized in various global and SSA settings [2, 9].

In Benin, recent research and implementation efforts to improve the quality of intrapartum care provision have been reported [20, 23–27]. On one hand, several studies on interventions promoting humanized care in Cotonou, the economic capital of Benin, found that midwives were initially hesitant to adopt RMC practices, such as assisting births in non-supine positions [25, 27]. On the other hand, one study found that implementing these care practices, over the long term, led to improvements in communication between midwives and patients, and improved midwives’ job satisfaction [27]. Explorations of the socio-cultural acceptability of RMC practices among all actors involved in the Beninese context are, however, are limited [21]. Given that childbirth is perceived as a time of physical, social, cultural and spiritual vulnerability in Benin [20, 21], developing an understanding of the acceptability of recommended RMC practices is critical. Investigating patient autonomy, as a relational concept fundamental to the provision of RMC, can provide insight into potential socially and culturally appropriate approaches for improving the quality of intrapartum care in Benin. To our knowledge, no studies have directly explored experiences of patient autonomy during childbirth in Beninese maternity wards. This study aimed to deepen the understanding of how women experience patient autonomy during intrapartum care through social practices in a Beninese maternity ward.

## Methods

### Study design

**Fig. 1:**
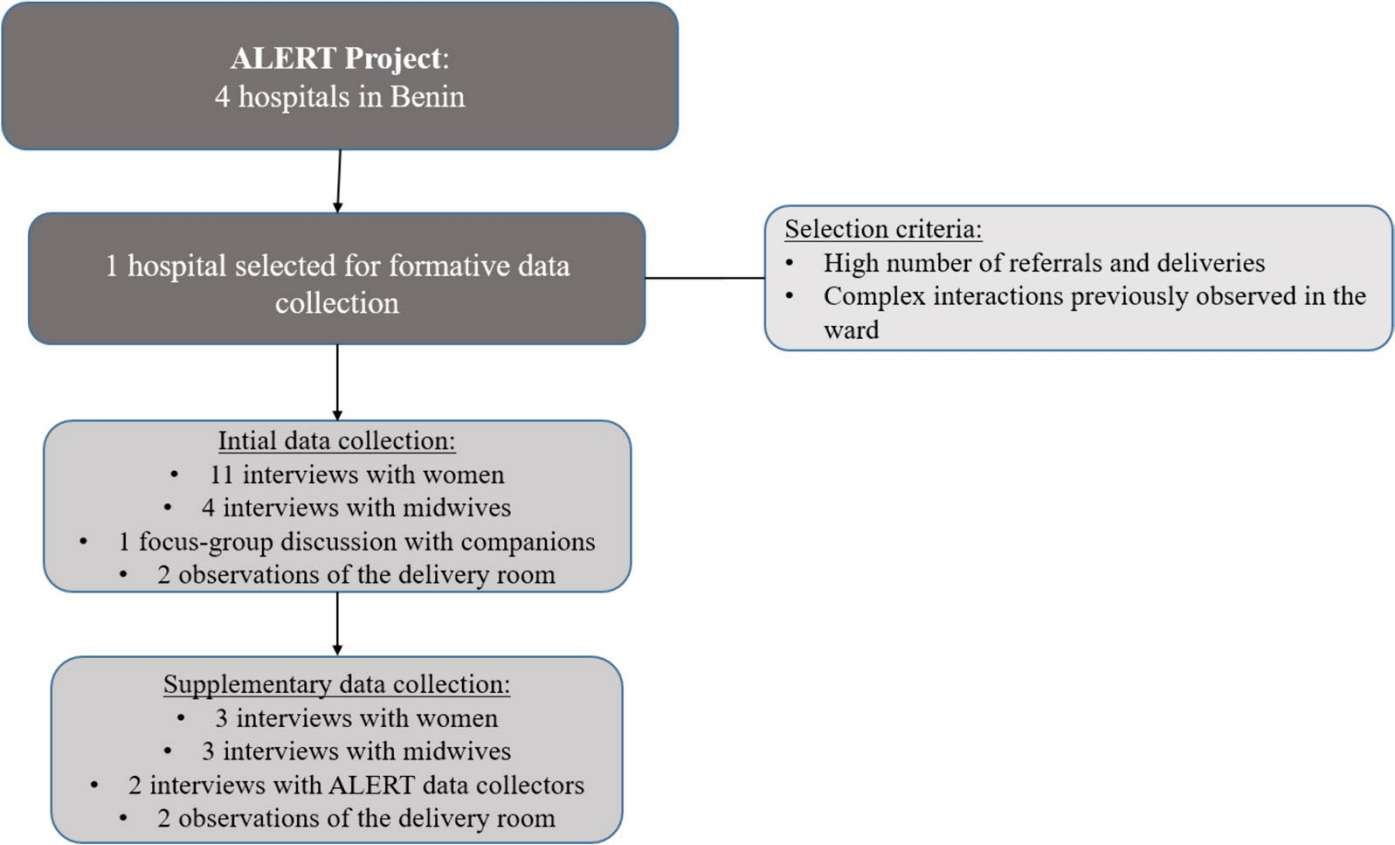
Facility selection and data collection process

This qualitative case study investigated manifestations of patient autonomy in a Beninese maternity ward. It was nested in the ALERT (Action Leveraging Evidence to Reduce Perinatal Mortality and Morbidity in Sub-Saharan Africa) research project, a multi-faceted, co-designed project aiming to achieve improvements in maternal and perinatal health outcomes at a health system level in Benin, Malawi, Tanzania, and Uganda [23]. Findings from initial data collection and co-design activities for ALERT intervention development were used and supplemental data was collected for the purpose of this study. (See Fig. 1). We included data from a public regional hospital in southern, inland Benin, currently participating in the ALERT project [23]. This hospital was purposefully selected for this study due to its high number of deliveries and referrals, in addition to the previously observed complex interactions between all actors involved during maternity care.

Semi-structured interviews with midwives and data collectors, a focus-group discussion (FGD) with women’s childbirth companions and non-participant observations of the delivery room were included, in addition to interviews with post-partum women (Fig. 1). This enabled us to gain a deeper understanding of the social practices present in the ward, and their influence on women’s experiences of patient autonomy during care.

### Setting

In Benin, although midwives and nurses are officially the main maternity care providers in institutionalized birth settings [28], only one midwife is available per 10 000 people [29]. With 80% of Beninese using “traditional” medicine, including during childbirth, there are six times as many “traditional practitioners” as physicians in the country [21]. Difficulties in access to healthcare have been, in part, attributed to distance to health facilities, reluctance to go seek healthcare alone, and needing permission from the head of their households to visit a doctor [28]. Since all healthcare-related costs are directly covered by the individual, the financial burden is another barrier to accessing care [28].

The public regional hospital selected is located between two large conurbations in a southern, inland department of Benin. The hospital offers caesarean section and blood transfusion services, has a minimum caseload of 2500 births per year [23], and receives many referral cases from peripheral health centres.

In 2016, 23.9% of the female population of the region was reported to be of childbearing age [30]. A majority (92.3%) belong to the Fon tribe, the largest ethnic group in the country [30]. Catholicism and Vodoun are the predominant religions in the region, although much religious diversity is present in the region and country as a whole [30]. Sixty-seven percent of the population in this region live in rural areas [30].

### Recruitment processes

Purposive sampling was used to identify post-partum women, midwives, companions and data collectors to participate either in semi-structured interviews or a FGD. Participants were asked in person by either AAV or GH, who are female and Beninese, to participate in the study. AAV and GH were actively involved in the ALERT research project, GH as a research assistant and midwife, and AAV as a sociologist. Interview and FGD participants were identified by observing dynamics in the maternity ward, and by having informal discussions with potential candidates about their experiences of care and negotiating their availability. Women who had undergone a caesarean section were approached on the day of discharge from the hospital. Women who gave birth vaginally, companions and midwives were recruited when available and willing to participate. Data collectors, GH and AAV, were recruited for interviews by the first author (NSRN) who is a female, non-Beninese Master’ graduate.

### Data collection

Initial interviews with women and midwives, the FGD with companions and observations of the delivery room were conducted from January to March 2021, during co-design and data collection activities for the development of the ALERT intervention. Eleven post-partum women were interviewed about their experiences of care, and four maternity care providers, all female, were interviewed on their experience of care provision, see Table 1. Seven companions, 2 male and 5 female, participated in a focus group discussion on care provision in the ward.

**Table 1:** Demographic characteristics of participants of initial interviews

Interviewee	Age	Educational level	Occupation	Religion
Woman post-partum	25	No formal education	Reseller	Muslim
Woman post-partum	30	No formal education	Reseller	Evangelical
Woman post-partum	27	No formal education	Reseller	Evangelical
Woman post-partum	28	Secondary	Reseller	Voudoun
Woman post-partum	18	Secondary	Reseller	Catholic
Woman post-partum	19	Primary	Tailor	-
Woman post-partum	25	No formal education	Seller	Evangelical
Woman post-partum	36	Secondary	Store owner	Muslim
Woman post-partum	23	Secondary	Tailor	Catholic
Woman post-partum	35	No formal education	Merchant	Voudoun
Woman post-partum	35	Secondary	Administration	Catholic
Maternity care provider	31	Midwifery degree	Midwife	Ekankar
Maternity care provider	42	Primary	Caregiver	Evangelical
Maternity care provider	44	Midwifery degree	Midwife	Catholic
Maternity care provider	41	Midwifery degree	Midwife	Evangelical

Topic guides for initial data collection interviews were standard to the ALERT project. They covered multiple aspects of women’s experiences of childbirth and care provision, and midwives’ practices and perspectives on care provision, including RMC. The FGD with companions covered their experiences of companionship, and interactions with midwives and women during care. Interviews and the FGD were conducted in a quiet room. With the permission of participants, interviews and the FGD were audiotaped. Interviews lasted approximately 30 min, the FGD took approximately one hour, and observations were conducted over a period of six hours. The observation guide was standard to the ALERT project, and covered the physical conditions, medical processes, and interactions in the delivery room. More details on these activities can be found in the ALERT intervention’s protocol [23].

The collected data were preliminarily analysed, and followed by supplementary data collection in March 2023, which comprised interviews with women, midwives and ALERT data collectors, as well as observations of the delivery room (See Fig. 1). Supplementary data collection activities aimed to explore, in more depth, the manifestations of patient autonomy in the ward that had been identified in the initial data set. Three post-partum women and three female maternity care providers were interviewed, see Table 2.

**Table 2:** Demographic characteristics of participants of supplementary interviews

Interviewee	Age	Educational level	Occupation	Religion
Woman post-partum	31	-	Hairdresser	Evangelical
Woman post-partum	21	-	Apprentice tailor	Evangelical
Woman post-partum	-	-	-	-
Maternity care provider	-	Midwifery degree	Midwife	-
Maternity care provider	39	Midwifery degree	Midwife	Catholic
Maternity care provider	46	Midwifery degree	Midwife/nutritionist	-

Topics for supplementary interviews covered the following: RMC, patient autonomy, and alternative care practices. Interview guides for supplementary data collection were developed in French for the purpose of this study. Supplementary interviews with women and midwives were led by NSRN who received support from GH to translate questions into the Fon language. During supplemental data collection, one midwife refused to participate in a recorded interview and one woman was not deemed to be in adequate physical condition to be interviewed. ALERT data collectors, GH and AAV, were interviewed by NSRN about their experiences and reflections over the course of initial data collection, as peer-checks. Interviews with women and midwives were conducted in a meeting room of the maternity ward, and with data collectors in their office space. With the permission of the participants, smartphone recordings were taken. Interviews lasted between 30 and 50 min. Supplementary observations were conducted over four hours, on two occasions, by NSRN, GH, and a male medical doctor involved in the ALERT project. Field notes were taken over the course of supplementary data collection.

### Data analysis

Reflexive thematic analysis, in line with Braun and Clarke’s six phases of thematic analysis [31, 32], was conducted using NVivo 1.7.1. We first analysed the initial data set. Interviews from the initial data collection were transcribed into French, but coded in English to facilitate the eventual translation of the results. The authors involved in coding (NSRN, AAV, CBH, HMA, RU and GH) are fluent both in English and French. Arrieta Valero’s five dimensions of patient autonomy (see Supplementary Table 1) were then used to guide the identification of initial codes [12, 14]. Initial codes were subsequently dissociated from the dimensions of autonomy to allow for more inductive analysis. Findings from the supplementary data collection, more specific to the concept of patient autonomy, were used to further refine initial codes, cluster them and develop preliminary themes. Mind maps were used to facilitate the clustering of codes and identification of themes. Final themes were established over the course of manuscript writing, in collaboration with all authors [32].

## Results

Two themes and five sub-themes were developed during thematic analysis, see Table 3*.*

**Table 3:** Themes and Sub-themes

Theme I: Patient autonomy is systemically suppressed because of the power imbalances present between women and midwives in the maternity ward	Sub-theme I: Care provision conditions do not favour patient autonomy
	Sub-theme II: Women experience acts of verbal and physical coercion during childbirth
	Sub-theme III: Women surrender their autonomy during intrapartum care
Theme II: Women use alternative and spiritual care practices to counteract experiences of limited patient autonomy during childbirth	Sub-theme I: The use of alternative medicine partially offsets the loss of patient autonomy during care
	Sub-theme II: Spirituality and religion are used as a coping mechanism during childbirth

### Theme I: Patient autonomy is systemically suppressed because of the power imbalances present between women and midwives in the maternity ward

#### Sub-theme I: Care provision conditions do not favour patient autonomy

Resource and infrastructure limitations influenced women’s experiences of intrapartum care. During initial data collection, the maternity ward had one delivery room with eight beds and no partitions, which negatively influenced women’s ability to maintain their privacy and confidentiality during birth. In between data collection points, a new delivery room was built. It contained 10 beds, separated by partitions, but with little space between them. In spite of improved privacy, women still encountered space constraints in the delivery room and bed shortages. Several participants described women giving birth on the floor. In some cases, this occurred due to the unavailability of beds.

*“Yes, and even the floor is saturated with women giving birth and it’s hard to move around the room. … Sometimes we find five women giving birth on the ground and that makes fifteen women in total. …” (Midwife_062).*

In other cases, women had a bed available but preferred lying on the floor, for the cool temperature. At the time of delivery, some women were no longer able to get back up onto the bed or wanted to stay in the same position, leading to them giving birth on the floor. Midwives expressed their willingness to support women giving birth on the floor, whether this was out of choice or necessity.

*“(Woman on the floor) … we tell her to get up and we help her get her on to the delivery bed. If she says no, we do everything on the floor. We stay squatted. It’s regrettable but it’s the rare cases that happen, we manage” (Midwife_068)*

Within the maternity ward, social practices also influenced women’s experience of autonomy during childbirth. Firstly, we observed that midwives spent very limited time with patients and had heavy workloads. Interviewed participants corroborated these observations.

*“… There were many of us. The providers went back and forth in the room to be able to serve each of us … I noticed that the number of nursing staff was not sufficient to support women giving birth, which leads them to work without rest”(Woman_053)*

The limited contact and emotional support from midwives influenced women’s opportunity to voice their preferences and decisions during care.

Secondly, many women described valuing the presence of a companion, who is comforting and supportive during intrapartum care. Hospital regulations, however, did not allow companions nor the use of cell phones in the delivery room, to maintain intimacy for other women. As described by the following companion, power imbalances in the ward limited the extent to which companions could advocate for women during care provision, even from outside the delivery room.

“*We did not come to complain … And even if we file a complaint, won’t that make them abandon our women? We risk making it difficult for them if we do.” (Companion_FGD_066).*

Thirdly, it was observed that pharmacological pain management was not systematically offered, but given only when very much needed. Midwives infrequently applied non-pharmacological pain management strategies, such as a massage or emotional support. It was observed that women had to manage pain and follow the course of childbirth mostly on their own.

Women, therefore, had limited decision-making opportunities, given the highly restricted support from midwives and companions, and a limited choice of pain management options.

#### Sub-theme II: Women experience acts of verbal and physical coercion during childbirth

Many midwives expected women to comply with their instructions during care provision. Verbal and physical violence was sometimes perpetrated in response to a lack of compliance. These acts were described by all participants to be committed mainly by midwives, but in some cases also by companions.

Yelling, shaming, discriminating, threatening, ridiculing, and ignoring were some of the forms of coercion described to occur during intrapartum care, and especially during the delivery.

*“There are times when the patient, despite everything we do, does not comply (to the instructions of midwives). This forces us sometimes to raise the tone or we call an accompanying person who will come do their part” (Midwife_062).*

Acts of physical violence, such as slapping and restraining, were also described in the ward.

*“(Q: In times when women don’t open their thighs during the delivery, how do you deal with it?) Two people keep the legs and one person ensures the delivery, so three people will attend a single woman’s delivery” (Midwife_06)*

In many cases, midwives justified these acts as being for the greater benefit of the woman and her child.

*“So we explain to the woman: ‘If you don’t want to let your baby go die, it’s up to you’. … So, the midwife said everything (possible), and she took a pillow to hit her so she would let herself go … and this is (now in) the court” (Midwife_068).*

These acts, not only infringed on women’s decisions during care, but also violated their bodies, and provoked feelings of fear in some. They, therefore, reinforced expectations of women’s compliance and hierarchical relationships between women and midwives during care, consequently limiting women’s ability to be autonomous during childbirth.

#### Sub-theme III: Women surrender their autonomy during intrapartum care

In light of the aforementioned limited decision-making opportunities and power imbalances, many women acknowledged consciously surrendering their autonomy during intrapartum care. Women described feeling uninvolved as actors in the process of intrapartum care, and feeling like care was mainly provided to their bodies. They often recounted that “their bodies no longer belonged to them” during the delivery, with one woman describing placing herself in the “hands of God and midwives”. Other participants mirrored this perception, describing women as having little control over their bodies and “letting themselves go” during intrapartum care.

In addition, many women acknowledged having limited knowledge of the intrapartum care they had received or needed, and described having inquired infrequently about their care at the time. The pain women experienced was expressed as one reason for their limited engagement during intrapartum care.

*“We are the ones who don’t ask or worry about anything, it’s not their (midwives) fault ...this is because many of us are suffering.” (Woman_054)*

All interviewee categories recognized women’s limited knowledge of medical processes during birth and of their rights during care provision as factors limiting their engagement.

*“They don’t know, they don’t know that they have the right to receive treatment or to say no to treatment … When they (women) come, they say, ‘Well I leave it to the person who knows his job. I give myself completely to her … I just want to have my baby and get out of here, that’s all’.” (Data collector_001).*

Midwives acknowledged that improved communication with women could increase their knowledge of institutional intrapartum care and improve woman-midwife collaboration. They expressed that this could, consequently, help them become more comfortable in voicing certain preferences or becoming more engaged in care provision during childbirth.

*“…She needs to be psychologically prepared … We must reassure the woman that we are all human; there is no fear here, if she has other worries there to tell us everything… Whatever she feels. Sometimes there are women who, in front of their husband, cannot express themselves well. In any case it is a moment when the woman must be given confidence.” (Midwife_069)*

### Theme II: Women use alternative and spiritual care practices to counteract experiences of limited autonomy during childbirth

#### Sub-theme I: The use of alternative medicine partially offsets the loss of patient autonomy during care

Both women and midwives mentioned that many women participated in alternative medical practices, such as attending traditional consultations and using local remedies, before, during and after intrapartum care, complementing the care provided in the health facility. Women generally described these experiences of alternative care as a positive, and even integral part of childbirth.

*“The fact that I gave birth safely and sound gave me a lot of pleasure. … the traditional consultations had revealed that I will give birth without a problem and that it is realized.” (Woman_055)*

*“(Q: … did you use something of faith (traditional herbal teas, prayer, etc.) before coming here?) I took ‘sugar’ (cubes) … She told me that I will find the strength” (Woman_056)*

Notably, midwives highlighted the use of “traditional oxytocin”, a plant-based traditional remedy in the form of tea, to accelerate childbirth. They described the use of these remedies as potentially having negative side effects, such as uterine rupture and haemorrhage. The consumption of these teas was prohibited at the maternity ward. All interviewee categories, however, mentioned that some women consumed them regardless of the restrictions. The secretive use of traditional remedies fuelled provider-patient mistrust.

*“(Q: When she starts to make those requests (to drink water or eat food) how do you react? … ) If she is hungry, then we will see, but drinking water is not systematically implemented because I don’t know what she is hiding.” (Midwife_068).*

#### Sub-theme II: Spirituality and religion are used as a coping mechanism during childbirth

As mentioned before, women experienced receiving limited support and pain management and having restricted autonomy during intrapartum care provision. We found that spirituality and religion were important ways for them to cope with these difficulties. They expressed relying strongly on their faith in God and their spirituality during childbirth. Women were regularly seen to pray and described themselves as having to pray as a way to cope with hardships encountered during childbirth, including experiences of pain, uncertainty, fear and loss.

*“I was scared, my heart was beating … (Q: And what did you do to endure until the end (of childbirth)?) It is God who saves us and I started by calling on God … Yes, and I started by praying.” (Woman_067)*

Women’s spirituality provided them with a sense of comfort, understanding and hope which helped them cope during intrapartum care.

*“It was God and the healthcare providers (who helped me during childbirth). Because God directs them for me… God gives them the wisdom to do whatever is best for me and my child. So I placed myself in the hands of God and the healthcare providers” (Woman_005)*

*“It was the work of God. When I arrived (there were no beds available), but someone had just finished giving birth. She was barely leaving and I arrived.” (Woman_067)*

In a couple of instances, women described speaking to or being comforted by their deceased father or unborn foetus.

*“I am proud of my religion … I was invoking the blessings and support of my father who passed away recently and was a great traditional man in our locality. …”* (W*oman_055)*

*“It was the foetus that comforted me… He was telling me to calm down that the doctors are coming soon and they are really coming to see me.” (Woman_065)*

Spirituality and religion were described by both women and midwives as a lens through which women could better understand the surrounding healthcare environment.

*“… put her at ease, tell her that the labour and the contractions of childbirth always hurt, and no one can change that. That it is natural, that it's God who did that and women must suffer childbirth.” (Midwife_069)*

In a healthcare context where women had little contact with and received limited emotional support from both midwives and companions, the use of spirituality and religion helped them accept the conditions of care provision.

## Discussion

### Main findings

Our study described various social practices reflecting patient autonomy during intrapartum care in a Beninese maternity ward, through the application of a multidimensional framework of patient autonomy [12, 14].

In spite of space limitations in the maternity ward, women who wanted to give birth on the floor were able to do so. Despite the midwives’ reluctance, given the discomfort of providing care to a woman on the floor, as seen in other instances [27], women were generally able to have decisional autonomy over their position of birth. Resource constraints in the delivery room did, however, make it difficult to maintain women’s privacy and confidentiality during childbirth. Renovations of the delivery room, conducted between data collection rounds, significantly improved privacy which, consequently, helped women maintain confidentiality during care. As certain procedures during care may be indicative of a woman’s health status, such as HIV status, improving privacy and confidentiality also improved women’s informational autonomy. Ensuring informational autonomy can lead to the provision of RMC by building trust during care and reducing the risk of stigmatization of a patient by midwives, other patients and companions [2, 3].

Midwives heavy workload in this study was found to limit the contact and emotional support women were provided, and limited their opportunities to voice their preferences and decisions during care. Reflecting the findings of our study, a Kenyan mixed methods study similarly reported that a large majority of healthcare providers recognized the importance of communication during maternity care and of women’s autonomy [8]. In practice, however, these values were not consistently upheld given pressure in the work environment, healthcare provider’s limited knowledge and skills, and women’s limited ability to assert their autonomy during care [8]. This restricted the ability of healthcare providers to successfully implement effective communication strategies [8], consequently, limiting women’s capacity to acquire the information needed to have decisional autonomy and hindering the provision of RMC [2, 3].

Women were in a position of physical vulnerability due to childbirth itself and the limited implementation of pain management strategies. They were also socially vulnerable given the power imbalances present between them and healthcare providers, and the limited emotional support available to them. In light of the limited support from midwives, companions could have contributed to positive childbirth experiences through the provision of physical, psychological and spiritual support, but were not allowed to, as has been demonstrated in other studies [3, 22, 33]. In other regions of SSA, hospital policies have similarly restricted companionship, and the consequent disempowerment of women during care has been demonstrated [34]. The absence of a companion in three other West African countries was also found to be associated with increased experiences of mistreatment, including physical or verbal abuse, stigmatization or discrimination [33]. Companions may, therefore, be able to prevent experiences of mistreatment, and advocate for the best interests of women [34], and thereby, improve women’s decisional and functional of autonomy during care. However, companions in our study were described as occasionally participating in mistreatment during care, upon request from healthcare providers. Companionship may, therefore, be beneficial to women’s experiences of birth as long as companions do not partake in coercive measures.

Pain, as described by Arrieta Valero, can influence all dimensions of autonomy by reducing the patient’s mental capacity to be autonomous [14]. Improving the management of women’s pain during childbirth could not only improve women’s decisional and functional autonomy during care, but would also ensure the provision of quality intrapartum care, in line with WHO standards [3].

Considering the physical and social vulnerability of women during intrapartum care and midwives’ expectation of compliant behaviour, the acts of coercion perpetrated in response to a lack of compliance reinforced power imbalances between women and midwives. Power imbalances, with similar accounts of verbal and physical abuse, discrimination, and poor rapport between women and midwives, have been reported by systematic reviews both on a global and SSA level [22, 34]. These highlighted that negative woman-midwife interactions, and systemic health system failures, consequently, disempowered women [22]. Similar to the findings of this study, in these systematic reviews, power was also described to be exerted through providers’ control over women’s bodies during birth [22, 34]. It was reported in South Africa, Ghana and Tanzania that healthcare providers would exert control over women’s bodies by restricting their natural urge to push during labour, and forcing them to push upon instruction [34]. Accounts from women in Ghana, Nigeria, Kenya and South Africa, echoed these findings, mentioning their fear of experiencing verbal or physical violence and receiving dehumanizing care in an institutional care provision context [35]. The use of verbal and physical coercive measures, has previously been described to infringe on women’s decisional and functional autonomy [14]. Disrespectful treatment, fear-based communication and negative experiences, such as non-consensual interventions, during antenatal care in Benin have previously been reported to act as barriers to seeking further antenatal care leading up to the delivery [36]. This highlights that the issue of RMC, autonomy and healthcare provider distrust extends beyond the perinatal period, and may lead to an increased risk of pregnancy complications if antenatal care visits are discontinued.

We believe that the power imbalances in the maternity ward and the control exerted over women’s bodies during care, as described here, may have been internalized by many women, reflected in them surrendering their autonomy or feeling uninvolved during institutional intrapartum care. In line with the findings of our study, a systematic review found that women in other SSA countries reported feeling like passive “bystanders” during care provision, due to gaps in information about care provision and limited support [34]. It has also, previously, been reported that women in southern Benin seek care at health facilities as late as possible, since women in more advanced stages of labour will receive care quicker than those in the initial stages [20]. By minimizing the time spent in the healthcare facility, women are limiting the time during which they have restricted autonomy over care. An emphasis on the need for promotion of women’s education and empowerment, to mitigate the healthcare system’s structural limitations, was highlighted by participants of this study, and has been previously reported in research from other SSA countries [8, 19]. Improving women’s education and empowerment would equip them with the knowledge and skills to acquire further information and vocalize their preferences about care, consequently improving their decisional, functional, informational, and narrative autonomy.

Childbirth has been described in some regions of Benin as a circular process of rebirth of ancestors, and a time requiring spiritual protection [21]. To ensure a successful delivery, women will attend consultations with the *Bokonon*, a wise man, partake in rites and rituals, and take herbal medicines, amongst other measures [21]. Mirroring our findings, alternative care practices have been previously reported to help women in Benin cope with diagnostic uncertainty and provide them with emotional support [21]. Although the incorporation of non-harmful alternative care practices could improve women’s experiences of care, and promote the provision of RMC and women’s autonomy during intrapartum care [2, 3, 14], their use is not always well accepted in institutionalized settings around Benin, and they are, therefore, commonly administered discreetly [21]. Working towards bridging the present disconnect between traditional medical practices and health facility intrapartum care could be beneficial to women’s wellbeing and increase the support offered over to women the course of the continuum of maternity care [20].

Women’s physical, social, cultural and spiritual vulnerability during pregnancy and childbirth generates much support from their family and community, in the form of physical protection, emotional support and spiritual protection [21]. Childbirth is a period of extensive uncertainty, vulnerability, and extreme pain. It is a situation in which they may seek spiritual and religious grounding. In this study, women used spirituality to cope with, even overcome, gaps in emotional support and breaches to their autonomy during care. Interestingly, in spite of the diversity in the religious beliefs of the delivering women in our sample, relying on religious beliefs and practices was widely described across the data set. It provided them with an internal sense of autonomy. Women may have resorted to their spirituality and religion for healing, previously described as using one’s own resources and values to achieve inner peace [37], during childbirth. These beliefs provided them with a lens through which to perceive, interpret and accept events that occurred during intrapartum care, which may have increased their narrative autonomy during care [12, 14]. This is crucial in developing respectful approaches to care, as narratively autonomous individuals will be able to exteriorize their expectations of childbirth and intrapartum care provision, and so, be able to better express their autonomy over their decisions and their body [12]. This study, therefore, identified spiritual autonomy as an emergent dimension of patient autonomy.

The literature has described the presence and pointed to the importance of spirituality during childbirth in Benin [21]. However, little is known about how to attend to women’s spiritual needs in an institutional birth setting [38]. Further research on incorporating spirituality into care is, therefore, needed in Benin, as a way to contribute to the promotion of RMC.

### Strengths and limitations

Our study contributed to the limited research on experiences of patient autonomy during intrapartum care in Benin. The inclusion of a variety of data collection methods and sources for triangulation enabled us to develop a comprehensive understanding of the manifestations of patient autonomy in the ward. Although the framework we used has been developed to understand patient autonomy during chronic care [12, 14], its application enabled us to look beyond decisional autonomy, to develop a relational understanding of patient autonomy during childbirth in the Beninese maternity ward. Reflexivity was established over the course of the study through multiple informal discussions between authors about the findings and the Beninese healthcare and social context.

There were some limitations in the data collection and analysis of this study. Firstly, interviews with women and midwives, and the FGD with companions were conducted in the maternity ward. This may have led participants to respond in a socially desirable way. Furthermore, non-participant observations in the delivery room may have led to a Hawthorn effect. Given previous collaborations and trust built with hospital management and healthcare providers over the course of the ALERT project, the risk of highly socially desirable responses and Hawthorn effects is lower than if no contact had been previously established. A second potential limitation in the data collection is that most of the interviews with women were translated first from Fon to French, and then to English, which may have resulted in losses of content and richness of the data. Reflexivity measures ensured the credibility of the results despite the multiple translations. Lastly, patient autonomy has most commonly been conceptualized as decisional autonomy [12], and experiences of patient autonomy have mainly been explored in the context of the Global North. Research on patient autonomy during intrapartum care is highly limited in the SSA context, although some publications have explored women’s autonomy (at household or community level) and healthcare usage [8, 16–19]. Many cultural, relational and contextual factors could limit the transferability of experiences of patient autonomy in Benin. Further research should explore women’s and healthcare providers’ conceptual understanding of patient autonomy during intrapartum care in Benin. This may contribute to developing context-relevant approaches for the provision of RMC in Beninese maternity wards.

## Conclusion

Our study highlighted the systemic suppression of women’s autonomy over the course of intrapartum care and demonstrated how alternative and spiritual practices were used to counteract this. Spiritual autonomy was identified as an emergent dimension of patient autonomy. This study calls attention to the importance of patient autonomy for improving the provision of RMC in Benin. Improving collaboration between biomedical and traditional care provision may improve women’s experiences of childbirth and promote the development of culturally appropriate and quality care provision.

### Supplementary Information

Supplementary Material 1.

## Data Availability

Data are available on reasonable request. All relevant data are within the manuscript and its supporting information files. There is no duplicity of data. The authors of this manuscript received access to the primary data set of the qualitative data from the Benin intervention sites of ALERT. Qualitative primary data, such as transcripts, reflect the views of women, healthcare providers and companions at a healthcare facility in southern inland Benin. Making the full data set publicly available could potentially breach the privacy that participants were promised upon request for participation. Also, our ethics approvals were granted based on the anonymity of the individuals consenting to participate. Due to these conditions, the authors are unable to share the full transcripts. Excerpts of specific segments of the text will be reviewed for any potentially identifying details and made available to fellow researchers or reviewers who complete a data sharing agreement and abide by strict confidentiality protocols. In line with the information given to the participants and restrictions set by the ethics committees above, access to the full transcripts is only available to the involved researchers. Data requests may be sent to the corresponding author, HMA, via helle.molsted-alvesson@ki.se.

## Abbreviations

ALERT: Action Leveraging Evidence to Reduce Perinatal Mortality and Morbidity in Sub-Saharan Africa
FGD: Focus-group discussion
RMC: Respectful Maternity Care
SRHR: Sexual Reproductive Health and Rights
SSA: Sub-Saharan Africa
