# Supplementary material for: “Letting themselves go during care” – exploring patient autonomy during co-designed intrapartum care in a Beninese maternity ward
Source: BMC Pregnancy Childbirth. 2024 Aug 30;24:566. doi: 10.1186/s12884-024-06777-5 (PMC11363672; doi:10.1186/s12884-024-06777-5)
Supplement: Supplementary file 1 — Supplementary Material 1. [file 12884_2024_6777_MOESM1_ESM.docx]

Supplemental Material

**Supplementary table 1:** Dimensions of Patient Autonomy as Used in this Study (14,16)

| Dimension of autonomy | Definition |
| --- | --- |
| Decisional | One’s ability to make clinically informed decisions free of coercion. Requires individuals to be adequately informed about the decisions being taken and the mental capacity to understand, value and express their choices. |
| Functional | One’s ability to conduct vital activities and self-govern one’s body (walking, seeing, eating, etc.). Requires the internal capacity to self-govern their body (physical, mental and sensory functioning) and the material possibility to exert their autonomy in their surroundings (i.e. limited functional autonomy may be due to internal factors or disabling environments). |
| Executive | One’s ability to plan, execute and maintain a certain therapeutic plan over time. (Especially relevant to adherence to treatment during chronic care provision and is increasingly important in the transition to patient-centred care.). |
| Narrative | One’s ability to understand, retain and communicate coherently the aspects which characterise them individually and aspects related to their medical condition. A narratively autonomous person will integrate a healthcare decision or situation into a shareable narrative of their illness and treatment. |
| Informational | One’s ability to manage and control their private and public information as desired. Relies on the processes in place for the management of personal information in a given context. (If one cannot access or control their private and public medical information, they will have limited self-governance.) |
